# Pancreatic carcinoma underlying a complex presentation in late pregnancy: a case report

**DOI:** 10.1186/s13256-018-1911-9

**Published:** 2018-12-15

**Authors:** Ai-Wu Shi, Xiao-Feng Shen, Hong-Juan Ding, Yao-Qiu Liu, Li Meng, Bill Kalionis

**Affiliations:** 1 Department of Maternity Intensive Care Unit, Nanjing Maternity and Child Health Care Hospital, Women’s Hospital of Nanjing Medical University, Nanjing, China; 2 Department of Maternal-Fetal Medicine Pregnancy Research Centre, Royal Women’s Hospital, Parkville, Australia; 3 Department of Obstetrics and Gynaecology, University of Melbourne, Parkville, Australia

**Keywords:** Gestational diabetes mellitus, Disseminated intravascular coagulation, HELLP syndrome, Pulmonary hypertension, Pancreatic cancer

## Abstract

**Background:**

Gestational diabetes mellitus is strongly related to the risk of pancreatic cancer in pregnant women, but gestational diabetes can precede a diagnosis of pancreatic cancer by many years. Women with a history of gestational diabetes showed a relative risk of pancreatic cancer of 7.1. Pancreatic adenocarcinoma is one of the most common malignancies associated with thromboembolic events. A clinical study showed that thromboembolic events were detected in 36% of patients diagnosed as having pancreatic cancer. Studies showed that gestational diabetes mellitus could be one of the important risk factors for pancreatic cancer.

**Case presentation:**

Gestational diabetes mellitus is associated with increased risk of breast and pancreatic cancer. This case report describes a 29-year-old Chinese woman who presented with: gestational diabetes mellitus; International Society on Thrombosis and Haemostasis criteria suggested disseminated intravascular coagulation with a score of 5; hemolysis, elevated liver enzymes, low platelet count syndrome; and pulmonary hypertension. After an intravenous injection of fibrinogen, she gave birth to a normal baby and following delivery, her blood pressure reached 180/110 mmHg. Laboratory analysis results showed elevated lactic dehydrogenase, decreased platelets and fibrinogen, and urine protein was positive. She was transfused with fresh frozen plasma, blood coagulation factor, and fibrinogen. Subsequently, she was transferred to a maternity intensive care unit, where magnesium sulfate seizure prophylaxis was continued for 24 hours to keep her magnesium level at a low therapeutic range. However, continuous oxygen therapy was needed to maintain her oxygenation. Further laboratory investigations revealed elevated carcinoembryonic antigen, carbohydrate antigen 19-9, and carbohydrate antigen 72-4. Positron emission tomography-computed tomography showed malignant carcinoma in the head of her pancreas with lymph node involvement along with bone, peritoneal, and left adrenal metastasis, as well as double lung lymphangitic carcinomatosis.

**Conclusion:**

A differential diagnosis of digestive system neoplasm should be considered when a pregnant patient presents with gestational diabetes mellitus and disseminated intravascular coagulation, where the disseminated intravascular coagulation has no specific cause and cannot be readily resolved.

## Introduction

Gestational diabetes mellitus (GDM) is strongly related to the risk of pancreatic cancer in this population but GDM can precede a diagnosis of pancreatic cancer by many years [1]. Women with a history of GDM show a relative risk of pancreatic cancer of 7.1 [1]. Pancreatic adenocarcinoma is one of the most common malignancies associated with thromboembolic events [2]. A clinical study showed that thromboembolic events had been detected in 36% of patients diagnosed as having pancreatic cancer [3]. These data suggest that GDM could be an important risk factor for pancreatic cancer. Here we report the case of a patient with pancreatic carcinoma underlying a complex presentation in late pregnancy. The case involves a 29-year-old Chinese woman who presented with: GDM; hemolysis, elevated liver enzymes, low platelet (PLT) count syndrome (HELLP); heart failure; and pulmonary hypertension. She was eventually diagnosed as having advanced pancreatic adenocarcinoma by positron emission tomography-computed tomography (PET-CT). She died 27 days after her natural delivery.

## Case presentation

A 29-year-old Chinese woman presented at 38 weeks’ gestation with GDM and was admitted to hospital awaiting delivery. Her blood test showed mild thrombocytopenia (PLT 73 × 10^9^/L). Her fibrinogen level (0.667 g/L) was decreased, while her anti-thrombin III (ATIII; 108.2%) and D-dimer (6.68 mg/L; normal range, 0–0.55 mg/L) levels were elevated. An evaluation of our patient according to International Society on Thrombosis and Haemostasis (ISTH) criteria (Table 1) [4] suggested overt disseminated intravascular coagulation (DIC) with a score of 5. After an intravenous injection of fibrinogen, her fibrinogen level reached 1.9 g/L and she delivered a normal baby girl weighing 3000 g. The Apgar score was 10–10 at 21:46 on the same day. One hour after her delivery, her blood pressure (BP) reached 180/110 mmHg.

**Table 1:** International Society on Thrombosis and Haemostasis diagnostic scoring system for disseminated intravascular coagulation. Scoring system for overt disseminated intravascular coagulation

Risk assessment: Underlying disorder	If yes: proceed If no: do not use this algorithm
Score the test results:	• Platelet count: > 100 × 109/L = 0; < 100 × 109/L = 1; < 50 × 109/L = 2 • Elevated fibrin marker (D-dimer*, fibrin degradation products): no increase = 0; moderate increase = 2; strong increase = 3 • Prolonged PT: < 3 seconds = 0; 3–6 seconds: = 1; > 6 seconds = 2 • Fibrinogen level: > 1 g/L = 0; < 1 g/L = 1
Calculate score:	≥ 5 compatible with overt DIC < 5 suggestive for non-overt DIC

*DIC* disseminated intravascular coagulation, *PT* prothrombin time. *D-dimer measurement: normal range as conventional units < 0.5 μg/mL (SI units: 0–3 nmol/L; nmol/L = μg/mL × 5.476)

Her physical examination results were as follows: temperature 36.8 °C, pulse 92 beats/minute, respiration 20 breaths/minute, and BP 180/110 mmHg; she was mentally healthy and moderately nourished. Her thoracic respiration was regular, and her chest expansion was symmetrical. Vesicular breathing sounds were normal and no moist rales were heard. Her heart rate was approximately 92 beats/minute with regular rhythm. There was no capillary pulsation and water hammer pulse, and no edema on either lower limb. Pathological reflexes were negative.

The laboratory findings were as follows: lactic dehydrogenase (LDH) 654.0 u/L, PLT 71 × 10^9^/L, fibrinogen 0.719 g/L, and urine protein (+). She was transfused with 400 mL of fresh frozen plasma, five units of blood coagulation factor, and 4 g of fibrinogen. She was then transferred to the maternity intensive care unit where magnesium sulfate seizure prophylaxis continued for 24 hours after the procedure. She was given the magnesium sulfate in small doses of 1–2 g to keep her magnesium level in the low therapeutic range. Her BP was steady and her coagulation function was improving but she needed continuous oxygen therapy to maintain oxygenation.

A chest X-ray showed evidence of lung edema. She had a history of a persistent cough and exertional asthma 4 weeks before she was admitted to hospital. She ignored these symptoms and consequently received no therapeutic intervention. There was no history of cardiovascular disease, family disease, or psychosocial disease.

Six days after hospitalization, her blood PLT count reached 96 × 10^9^/L, her fibrinogen level was increased to 1.784 g/L, and D-dimer was also elevated (9.51 mg/L). Her prothrombin time (PT), activated partial thromboplastin time (APTT), and ATIII were all normal. However, she constantly needed oxygen inhalation at 33% oxygen concentration to maintain oxygenation and her hypoxemia was difficult to control. Laboratory investigations revealed elevated carcinoembryonic antigen (CEA), (24.4 ng/mL), carbohydrate antigen (CA) 19-9 (> 1000 U/mL), and CA72-4 > 300 U/ml. A transthoracic echocardiography (TTE) examination showed normal dimension of the atrium and ventricle, but decreased left ventricular function. The left ventricular ejection fraction was 55%. Moderate tricuspid regurgitation with a peak velocity of approximately 3.56 m/s was recorded. The estimated pressure of the pulmonary artery from the tricuspid regurgitation was approximately 56 mmHg. Arterial and venous Doppler ultrasonography was performed on both legs and the results were normal. PET-CT showed malignant carcinoma of the head of the pancreas with lymph node involvement, bone metastases, peritoneal metastasis, bilateral lung cancer lymphangitis, and left adrenal metastasis. She died 2 weeks after the diagnosis before the scheduled initiation of chemotherapy.

## Discussion

Studies showed that patients with a history of GDM have an increased risk for future breast, ovarian, and uterine malignancies [5]. On the other hand, large population-based studies did not find a greater risk of cancers among women with GDM during the first decade postpartum. However, GDM was associated with a higher risk of thyroid cancer and a lower risk of premenopausal breast cancer over a median 8-year follow-up [6]. The prevalence of GDM is reported to be increasing [7] and is more common among African-Americans, Hispanics, Asians, and Native Americans than among non-Hispanic whites [8–10]. In this case, we reported the case of a woman with GDM with HELLP syndrome and DIC, who was eventually diagnosed as having advanced carcinoma of the pancreas.

This case is an unusual pancreatic carcinoma underlying a complex presentation of GDM, HELLP syndrome, pulmonary hypertension, and DIC in late pregnancy. Differential diagnoses included acute fatty liver of pregnancy, thrombotic thrombocytopenic purpura (TTP), and hemolytic uremic syndrome (HUS). In pregnancy and postpartum, thrombotic microangiopathy (TMA) is most commonly encountered in patients with HELLP or severe preeclampsia, but more rarely TMA is due to TTP or HUS [11]. Due to overlapping clinical and laboratory features, TTP and HUS are often mistaken for HELLP or preeclampsia. HUS and TTP are two microangiopathic disorders described in case reports and small case series with an estimated incidence of 1 in 100,000 pregnancies. The triad of HUS is classically described as thrombocytopenia, microangiopathic hemolytic anemia, and renal dysfunction. The addition of fever and neurologic symptoms makes the classic pentad of TTP [12]. In this case, a physical examination of our patient revealed hypertension but no evidence of purpura or petechia, neurologic abnormalities, or edema. Her deep tendon reflexes were normal.

Broken red blood cells were seen in the peripheral blood smear. The urine analysis studies revealed a + 1 proteinuria, PLT 73 × 10^9^/L, fibrinogen 0.667 g/L, and a normal creatinine level. According to her presentation, physical examination findings, and laboratory results, we made a differential diagnosis of microangiopathic hemolytic anemia in late pregnancy. From the outcome of this case, we recommend a multidisciplinary team approach (Critical care, Hematology, Maternal Fetal Medicine, and so on) to expedite diagnosis and treatment, which may be lifesaving.

Among patients with pancreatic cancer, approximately 40% develop diabetes in the 36 months preceding the diagnosis of pancreatic cancer. Normal pancreatic cells metabolize glucose by oxidative phosphorylation, whereas pancreatic cancer cells mainly metabolize glucose through aerobic glycolysis. This process provides less energy but more metabolic intermediates, which are required for sustaining cell proliferation and thereby providing an advantage in survival, to counterbalance deficient energy production and meet the increasing growth needs of pancreatic cancer cells. Thus, diabetes may provide additional glucose that supports pancreatic cancer cell function. A meta-analysis showed that diabetes was the strongest risk factor for developing pancreatic neuroendocrine tumors with an odds ratio (OR) as high as 2.74 [13]. Other studies also showed diabetes was found to be associated with an increased risk of pancreatic neuroendocrine tumors [14, 15]. In HELLP syndrome, pregnancies may be complicated by intrauterine growth retardation, premature delivery, placental abruption, respiratory distress syndrome, and cerebral hemorrhage in the neonate. In this case, our patient presented with HELLP, GDM, DIC, and unrecoverable pulmonary hypertension. Finally, she was diagnosed as having advanced pancreatic cancer. We had not considered this outcome. After reviewing the literature, we found no reported cases that were similar. Therefore, this case may prove valuable in strengthening the communication between doctors and assist in diagnosis and prognosis.

Our patient died suddenly, 2 weeks after the diagnosis of pancreatic carcinoma and appeared to have died from a pulmonary embolism. Unfortunately, we did not perform an autopsy, which is also a limitation of this case report. From this case it remains unclear whether GDM was a risk factor for the occurrence of pancreatic tumor or whether this association is a secondary effect related to the pancreatic tumor. Early detection of impaired glucose tolerance or diabetes may drive patients to change their lifestyle which includes reducing weight, changing their diet, and increasing physical activities. These changes are likely to improve blood glucose metabolism, which may be an effective way of avoiding, delaying, or reducing pancreatic cancer.

## Conclusions

In this case report, we described a patient with GDM, HELLP syndrome, heart failure, and pulmonary hypertension in late gestation, who was eventually diagnosed as having advanced pancreatic adenocarcinoma by PET-CT. A differential diagnosis of digestive system neoplasm should be considered when a pregnant patient presents with GDM and DIC, where the DIC has no specific cause and cannot be readily resolved.
